# Monitoring of single extracellular vesicle heterogeneity in cancer progression and therapy

**DOI:** 10.3389/fonc.2023.1256585

**Published:** 2023-09-26

**Authors:** Yoon-Jin Lee, Shinwon Chae, Dongsic Choi

**Affiliations:** Department of Biochemistry, College of Medicine, Soonchunhyang University, Cheonan, Chungcheongnam, Republic of Korea

**Keywords:** extracellular vesicle, EV subtype, heterogeneity, chemotherapy, single EVs, liquid biopsy

## Abstract

Cancer cells actively release lipid bilayer extracellular vesicles (EVs) that affect their microenvironment, favoring their progression and response to extracellular stress. These EVs contain dynamically regulating molecular cargos (proteins and nucleic acids) selected from their parental cells, representing the active biological functionality for cancer progression. These EVs are heterogeneous according to their size and molecular composition and are usually defined based on their biogenetic mechanisms, such as exosomes and ectosomes. Recent single EV detection technologies, such as nano-flow cytometry, have revealed the dynamically regulated molecular diversity within bulk EVs, indicating complex EV heterogeneity beyond classical biogenetic-based EV subtypes. EVs can be changed by internal oncogenic transformation or external stress such as chemotherapy. Among the altered combinations of EV subtypes, only a specific set of EVs represents functional molecular cargo, enabling cancer progression and immune modulation in the tumor microenvironment through their altered targeting efficiency and specificity. This review covers the heterogeneity of EVs discovered by emerging single EV analysis technologies, which reveal the complex distribution of EVs affected by oncogenic transformation and chemotherapy. Encouragingly, these unique molecular signatures in individual EVs indicate the status of their parental cancer cells. Thus, precise molecular profiling of circulating single EVs would open new areas for in-depth monitoring of the cancer microenvironment and shed new light on non-invasive diagnostic approaches using liquid biopsy.

## Introduction

Extracellular vesicles (EVs) are lipid bilayer particles ranging from 30 nm to 1 µm in diameter (1). These nanoscale vesicles are released from most cell types into the extracellular space and surrounding biological fluids (1). Consequently, EVs exist in all body fluids, including the blood, urine, tears, saliva, and cerebrospinal fluid, and are involved in both local and long-range communication through the regulated exchange of cellular materials (2). However, cancer cells and their neighboring cells in the tumor microenvironment dynamically regulate EV release in response to extracellular conditions, such as hypoxia, inflammation, or therapeutic stress (1, 3). These secreted EVs are involved in almost all aspects of malignant progression, such as cellular survival and environmental remodeling, including the generation of vascular networks, thrombosis, and inflammatory regulation (3). This multi-functionality of EVs depends on their complex molecular components such as proteins, genetic materials (mRNA, miRNA, snRNA, and DNA), metabolites, and lipids in different EV subtypes generated by diverse biogenesis mechanisms (3). Currently, the umbrella term EV has been widely used to cover all EV subtypes defined by the International Society for Extracellular Vesicles (ISEV), classified as exosomes, ectosomes, microvesicles, shedding vesicles, oncosomes, and other terms defined by their biogenesis mechanism, source of parental cells, or functionality (4). Although the morphological features of these EV subtypes are similar in vesicular shape under submicron size, they are not uniform in terms of size and molecular composition in each EV (2, 5).

Regarding biogenesis, EVs are broadly divided into exosomes and ectosomes (**Table 1**) (18). Simply put, exosomes originate from endocytic vesicles in multivesicular bodies (MVBs) with smaller EVs ranging from 30 nm to 150 nm, but ectosomes, known as microvesicles, shed larger EVs from the plasma membrane over 100 nm up to 1 µm (18). Both EV subtypes are released together in the same cell, but some cells preferentially release one type of EV depending on specific extracellular stimuli (e.g., epidermal growth factor receptor (EGFR)) or cellular transformation derived from oncogenes (e.g., RAS and EGFRvIII) (2, 6). Other EV types, such as arrestin domain-containing protein 1-mediated microvesicles (ARMMs) and apoptotic vesicles, are also categorized as EVs (13–15). Recently, EV-like membrane-less particles, such as exomeres, supermeres, and chromatimeres, were identified (9, 17, 19). They have similar vesicular characteristics with sedimentation at high g-force and share molecular composition, including proteins and RNAs, but their physiological nature regarding lipid structure has not been well studied.

**Table 1:** Subtypes of EVs and membrane-less particles.

Name	Size	Markers	Isolation	Subcellular orientation	References
Exosomes	30–150 nm	ALIX, TSG101, syntenin-1, and LAMP1	1.075–1.125 g/mL; 100,000 × g sedimentation and iodixanol density gradient ultracentrifuge	MVB	(6–8)
Ectosomes (i.e., shedding vesicles and microvesicles)	100–1,000 nm	ARF6, annexin A2, and BSG	1.090–1.115 g/mL; 10,000 × g sedimentation and iodixanol density gradient ultracentrifuge	Plasma membrane	(6, 7, 9–12)
ARMMs	40–100 nm	ARRDC1 and TSG101	120,000 × g sedimentation	Plasma membrane	(13)
Apoptotic vesicles or blebs	50–5,000 nm	Histones, DNA, and phosphatidylserine	1.16–1.28 g/mL; iodixanol density gradient ultracentrifuge	Nucleus and intracellular organelles in apoptotic cells	(14, 15)
Exomeres	~35 nm	HSP90AB1 and metabolic enzymes	Asymmetric flow field-flow fractionation	Unknown	(16)
Supermeres	<35 nm	TGFBI and GPC1	367,000 × g sedimentation	Unknown	(17)
Chromatimeres	<200 nm	DNA	Size exclusion chromatography	Unknown	(9)

ARMMs, arrestin domain-containing protein 1-mediated microvesicles; MVB, multivesicular body.

In reality, each EV subtype categorized as exosomes or ectosomes is composed of individual vesicles with different physiological properties and compositions, although they have similar biogenesis mechanisms. Thus, the current EV subtype definition may not represent the distinctive characteristics and functionality of individual EVs (2, 5). Tetraspanins, including CD9, CD63, and CD81, have been widely studied as canonical markers enriched in EVs (14). Initially, it was believed that these tetraspanins co-exist in an EV released from the cell, in which they are clustered together with other accessory proteins such as integrins in the cells, forming a tetraspanin web (20). However, many studies have suggested that rather than all together in a single EV, different combinations of tetraspanins (e.g., CD63 only and both CD63 and CD9) can be found in an EV (7, 9, 21). Importantly, recent single EV analysis technologies, such as single vesicle imaging by super-resolution microscopy or detection by nano-flow cytometry, have revealed a heterogeneous mixture of individual EVs defined by antigens secreted from the cells (9, 22). This evidence suggests that cells generate a collection of distinctive EVs with different surface antigen decorations. Although these EV subsets seem to partially overlap in their molecular composition, their unique combination enables distinctive functionality and target specificity (23). For example, Hoshino et al. reported the differential tissue-targeting specificity of integrin α6β4-positive EVs for lung tropism and integrin αvβ5-positive EVs for liver tropism (23). Moreover, EV corona and surface decoration by non-integral membrane proteins (e.g., fibronectin) on EVs could determine the uptake efficiency of each EV (24, 25).

In particular, cancer cells actively release diverse types of EVs as well as increased numbers of total EVs (9). This complexity of EVs derived from cancer cells is largely affected by internal oncogenic mutations, including EGFR, HER2, AKT, SRC, and RAS (2). In addition, cancer cells modulate their EV release in response to therapeutic stress, such as chemotherapy, for survival and drug resistance (26). These alterations in EVs affected by cancer progression and chemotherapy have been considered potent biomarkers for the diagnosis and prognosis of cancer (27). In addition, treatment with chemotherapeutic drugs elicits the release of EVs, inducing metastasis and subtype change (9, 26). Thus, the examination of circulating EV heterogeneity can provide direct information on therapeutic responses in cancer patients, including therapy resistance (27, 28). In this review, we summarize the classical EV subtypes and other subtypes revealed by single EV analyses and provide an overview of the current methods to analyze the subtypes of circulating EVs at the single vesicular level and their subtype change during cancer progression and chemotherapy.

## Classical EV subtypes: exosomes and ectosomes

**Figure 1:**
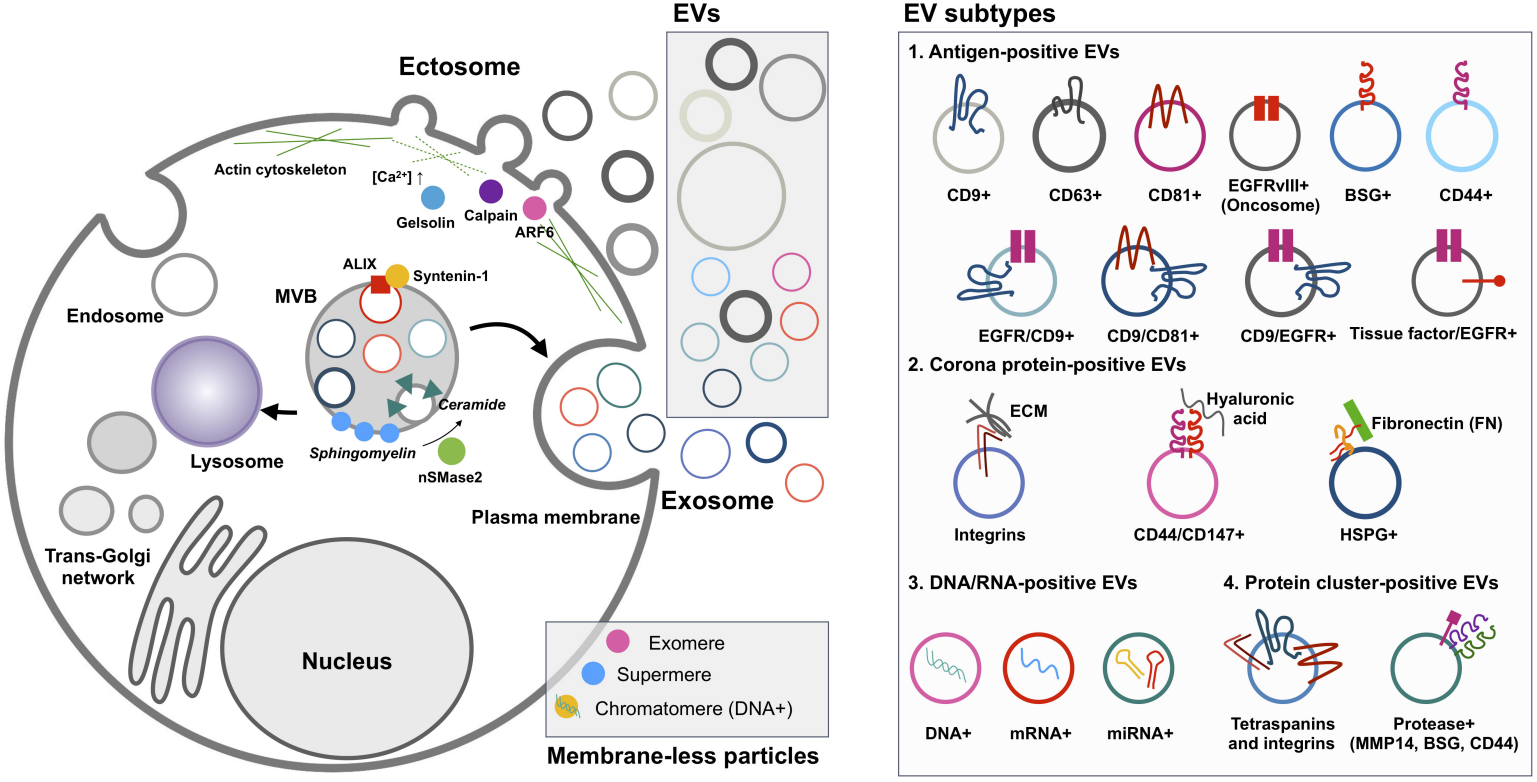
EV biogenesis and heterogeneity. Cancer cells release ectosomes, exosomes, and membrane-less particles such as exomeres, supermeres, and chromatimeres together. However, their complexity is much greater in terms of molecular composition in individual EVs. Oncogenic transformation influences the change of individual EV phenotypes related to tumor aggressiveness and functionality in the tumor microenvironment. For example, the differential tissue-targeting functionality of integrin α6β4-positive EVs for lung tropism and integrin αvβ5-positive EVs for liver tropism was reported (23). Surface decoration by fibronectin on EVs could determine the uptake efficiency of each EV (24, 25). EV, extracellular vesicle.

Major classes of EVs are categorized based on their biogenesis mechanisms, such as exosomes and ectosomes (known as microvesicles or shedding vesicles) (**Figure 1**) (18). Smaller EVs, referred to as exosomes, range from 30 nm to 150 nm and originate from endosomal MVBs. Historically, exosomes were first observed by the Stal and Johnstone groups in 1983 using transmission electron microscopy during reticulocyte maturation to remove transferrin receptors by exosomes (29, 30). They observed the fusion of MVBs to the plasma membrane, resulting in the release of intraluminal vesicles into the extracellular space. Impressively, recent advanced live imaging technology was able to visualize MVB events on the plasma membrane with the release of exosomes (31). Exosomes are enriched with specific proteins, including ALIX (programmed cell death 6-interacting protein, PDCD6IP as official gene symbol), TSG101, and syntenin-1 (SDCBP). These proteins are related to the endosomal sorting complex required for transport (ESCRT) machinery for the generation of intraluminal vesicles, accompanied by the sorting of ubiquitinylated cargo into intraluminal vesicles in MVB (32). In particular, ALIX, TSG101, and syntenin-1 are highly enriched non-integral membrane proteins, which are distinctive characteristics of exosomes from other EV subtypes enriched with integral membrane proteins, including tetraspanin CD9, CD63, and CD81. ALIX, known as an ESCRT-associated protein, recruits ESCRT-III proteins to endosomes, enabling protein sorting into intraluminal vesicles in MVBs (33). During this process, syntenin-1 interacts with ALIX and supports the intraluminal budding of endosomal membranes (32).

Ectosomes, larger EVs, range from 100 nm to 1,000 nm and are shed from the plasma membrane through the detachment of their budding site (18). The first ectosomes were observed in 1946 as clotting factors in the blood by Chargaff and West (34), and later Wolf in 1967 described these membrane fragments that were derived from platelets (35). Ectosome biogenesis mechanisms have not been as well addressed as exosomes, but their release seems to be related to the activation of signaling pathways. For example, mutant EGFRvIII in glioma cells stimulates EGFRvIII-carrying ectosome, termed an oncosome, release with horizontal transfer of oncogenic receptor, which can merge with the plasma membrane in recipient cells lacking EGFRvIII, leading to the activation of EGFR-regulated signaling pathways such as MAPK/ERK (36). In addition, activating cells by an increased level of intracellular Ca^2+^ induces shedding EV generation (37). Increased Ca^2+^ concentration in the cell drives symmetric phospholipid distribution by scramblase and floppase translocating phosphatidylserine and phosphatidylethanolamine to the outer side of the plasma membrane (37). Consequently, the activation of Ca^2+^-dependent proteases, including calpain and gelsolin, induces degradation of the cytoskeleton, enabling budding of the membrane (37). Specifically, phospholipase D and ERK activate the GTP-binding protein ARF6 and myosin light chain kinase for ectosome release in invasive cells (10). In particular, prostate cancer cells shed large EVs, known as large oncosomes, ranging from 1 µm to 10 µm, and this large ectosome blebbing is promoted by oncogenic SRC activation (38). Exclusive marker proteins for ectosome populations are very limited, but plasma integral membrane proteins such as integrins and EGFR and cytosolic proteins including actin and GAPDH are relatively enriched in ectosomes compared to other EV subpopulations (9). Jeppesen et al. defined annexin A1 as a specific ectosomal marker protein based on the isolation and proteomics of EVs by high-resolution density gradient ultracentrifugation and observation of the budding of annexin A1-positive plasma membrane regions (8).

## EV heterogeneity and subtypes

**Figure 2:**
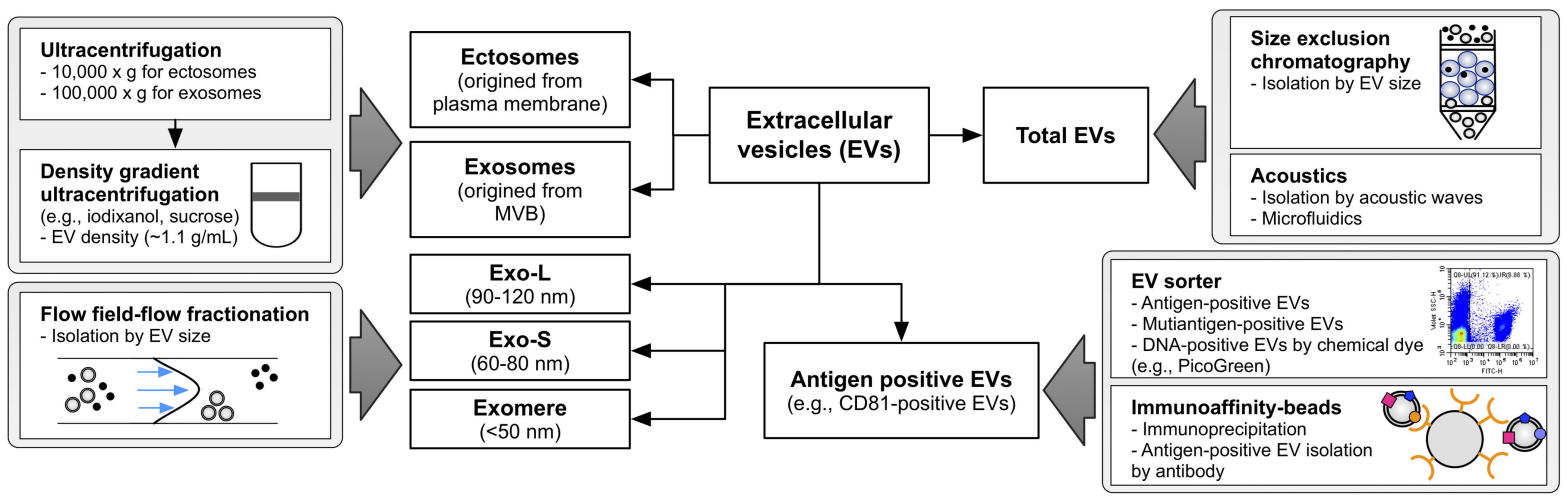
Overview of methodological approaches to isolate EV subtypes. Differential centrifuge and density gradient ultracentrifuge are widely used in exosome and ectosome enrichment based on their unique density. Flow field-flow fractionation effectively divides the EVs according to size. Size exclusion chromatography and acoustics could be applied to isolate the total EVs. Immunoaffinity purification and recent EV sorting technology are able to enrich the specific antigen-positive EVs. Exo-L means large exosome vesicles (approximately 90–120 nm), and Exo-S means small exosome vesicles (approximately 60 nm) as defined by Zhang et al. (19). EV, extracellular vesicle.

EVs are sedimented at high centrifugation forces over 100,000 × *g* for exosomes and 10,000 × *g* for ectosomes (9) and floatation at specific densities of 1.11–1.19 g/L of sucrose or iodixanol gradients (6, 11, 12). Based on these unique characteristics of EVs, their subtypes have been extensively addressed in previous studies based on size and density (**Figure 2**) (6, 8, 19). Asymmetric flow field-flow fractionation, which resolves mixed EVs based on their size, has revealed distinctive subtypes of EVs, including large exosome vesicles (Exo-L) (90–120 nm), small exosome vesicles (Exo-S) (60–80 nm), and membrane-less particle exomeres (approximately 35 nm) (19). Impressively, larger exosomes show an increased negative zeta potential, but the exomere has a less negative zeta potential (19). Large and small exosome vesicles seem to be similar to each other, but large subtypes are more equipped with ESCRT machinery involving the classical exosome biogenesis pathway, whereas small exosomes are enriched with lipid raft-related flotillin proteins, which are related to ESCRT-independent exosome biogenesis (16). However, exomeres showed a more unique proteomic composition related to glycolysis and the mTORC signaling pathway with the functionality to transfer their cargo to the recipient cells (16, 19).

Kowal et al. revealed proteomic differences between ectosome and exosome subtypes sedimented at 10,000 and 100,000 × *g*, respectively (6). Although this separation showed a distinctive size difference between the two populations, proteomic compositions overlapped with each other (6). Partially, endosome-related proteins such as EH domain-containing proteins (EHDs) and syntenin-1 are relatively enriched in exosome subpopulations, but cytoskeleton proteins, including actinins, are enriched in ectosome fractions. To precisely isolate EVs according to their density, Jeppesen et al. applied density gradient ultracentrifugation to isolate a pure exosome population, termed small EVs, from other EVs (8). The EV proteomes showed enriched ESCRT-related proteins such as TSG101, ALIX, and syntenin-1, but depletion of ribosomal proteins, histones, and extracellular matrix proteins, which are considered contaminants or EV-membrane attached proteins (39), as in other proteome studies of EV isolated by density gradient ultracentrifugation (8). Consistently, these studies showed that small EVs are mainly composed of exosome subsets with enrichment of plasma membrane, endosome, and ESCRT-related proteins but depleted of other subcellular organelle proteins.

Although these EV size- and density-based sub-fractionations effectively isolated EVs from non-vesicular contaminants such as protein aggregates or non-membranous particles, their subpopulations defined by homogeneous molecular composition still overlap with each other, implying that the molecular heterogeneity of EVs is more complex over these fractionations. The expected diversity of EVs is inferred from the number of identified molecules in the OMICS analyses. For example, EV proteomic analyses have usually revealed 1,000–3,000 distinct proteins from viable and relatively uniformly cultured cancer cells (3, 6, 8). If the diameter of the exosome is approximately 100 nm, only several hundred membrane proteins could be displayed (40), and the cellular synaptic vesicle, near-exosomal size, possibly contains approximately 200 proteins in a single vesicle (41). This calculation estimates that cells release several to hundreds of different subtypes of distinctive EV with non-overlapping proteomic composition (3, 20).

## Current advances for single EV analyses

While bulk EV isolation and its functionality provide valuable biological knowledge, increasing evidence suggests that the underlying heterogeneity within the bulk EV is far more complex (2). Recently, EV subpopulations have become accessible using advanced separation approaches, such as asymmetric flow field-flow fractionation (19), fluorescence-activated vesicle sorting (FAVS) by flow cytometry (42), and acoustic microfluidics (43). In addition, the physical properties of a single EV are applied to detect antigen-carrying EV subsets using surface plasmon resonance (44) and Raman spectroscopy (45) with high sensitivity. In addition, relative quantitation to monitor antigen-positive EVs has been achieved with antibody-based high-throughput methods, such as improved ELISA and immunocapture assays (46), array-based EV detection technologies (47), and multiparameter chip-based microfluidics (48).

Because of their submicron size, especially exosome subpopulations ≤100 nm diameter, EVs are below the detection thresholds of most standard optical imaging methods (e.g., approximately 250 nm in confocal microscopy) (49). Thus, their individual characteristics and diversity have long remained elusive (2). Subsequently, several EV measuring technologies for counting and sizing were able to characterize single EVs, such as electron microscopy (EM), atomic force microscopy, nanoparticle tracking analysis (NTA), dynamic light scattering (DLS), and tunable resistive pulse sensing (TRPS) (2). Typically, transmission electron microscopy (TEM) and cryo-electron microscopy (cryo-EM) are considered the gold standard technologies to directly observe the EV distribution for the validation of other indirect measurements and characterization methods (4).

Single EV imaging analysis could provide the anatomical view of an individual EV. Structured illumination microscopy (SIM) discriminates the distinctive subpopulation of CD63-positive and CD81-positive EV from CD63/green fluorescent protein (GFP)-expressing A431 cells (9). In this study, GFP tagged in the cytoplasmic domain of CD63 is located on the luminal side of EV, but anti-CD63 antibody fluorescence is located on the EV surface (9). Impressively, direct stochastic optical reconstruction microscopy (dSTORM) provides a more detailed view of EVs by super-resolution image with approximately 20-nm resolution through the time-resolved localization with sequential activation of fluorophores, which switches a non-fluorescent dark state to transient activation stochastically (50). Higginbotham et al. applied the STROM to reveal the colocalization of EGFR and CD9 in an individual sorted EV by flow cytometry (42). McNamara et al. visualized hundreds of individual EVs in a field of view by dSTORM from ONI Nanoimager, providing the uneven localization of tetraspanin CD81 on an EV labeled by lipophilic dye cell mask (51). This result implies that EV contains the distinctive membrane microdomains or lipid raft with the clustered tetraspanin proteins (51).

However, super-resolution imaging and EM technology are not available for high-throughput quantitative estimation at the single vesicular level, and EM methods are technically inconsistent. NTA-, DLS-, and TRPS-based methods effectively quantify the concentration of EVs with size information. However, these are not appropriate for quantitation of the specific subpopulation of specific antigen-carrying EVs. NTA could provide the fluorescent mode to detect specific EV subtypes labeled by fluorescent chemicals or antibodies, but their long recording time makes it difficult to record stable fluorescent-positive particle movement due to photobleaching (52). Impressively, recent advanced technologies are capable of molecular phenotyping single EVs using super-resolution microscopy (42), imaging flow cytometry (53), high-resolution flow cytometry (42, 54, 55), interferometric imaging (56), and a single EV capture platform on a chip (48). These high-throughput analyses of single EVs enable the decoding of the heterogeneity and broad molecular spectrum of EVs, even if released from a single cell line (57).

## Single EV analyses by nano-flow cytometry

There has been significant progress in nano-flow cytometry, known as high-resolution flow cytometry (5), enabling the detection of a single EV with multiple parameters (e.g., size and molecular composition) and high sensitivity (**Table 2**) (64). To detect and resolve the submicron-sized EVs, the instruments have adopted several improvements such as the high-sensitivity photomultiplier tube detector, avalanche photodiode detector, fluorescence triggering, detection of unique angles of light scattering, low-wavelength laser for side scattering, and software improvements (42, 54, 58, 65). In particular, low-wavelength lasers, such as the violet laser (405 nm), showed better sensitivity and resolution for small (100–500 nm) particles than the widely used blue laser (488 nm) in side scattering (65). This nano-flow cytometry analysis for a single EV detection represents the non-homogeneous EV subsets based on surface proteins (**Figure 1**) (55, 57, 58). This remarkable difference in surface antigen distribution in each EV could be of great significance in the context of EV-biomarker application, where the molecular context of each EV is equipped with valuable distinctive diagnostic information about the parental tumor or host cells from which they are derived as well as EV target specificity and functionality (23). Higginbotham et al. revealed the selective release of conformationally active EGFR-positive EVs from human colorectal cancer cells in the plasma of a mouse animal model and human patients (42). Moreover, single EV distribution is dynamically regulated, reflecting its parental cellular status in the tissue microenvironment, similar to the regulation in single-cell transcriptomics of cancer and normal tissues (2). As a result, biological fluids contain differential properties of diverse EV subtypes from various cells with different physical and molecular properties.

**Table 2:** Identification of EV subtypes by nano-flow cytometry.

Cells or biological fluids	Nano-flow cytometry	EV isolation	EV labeling	Target antigen	Reference
Human platelet-free plasma	Beckman Gallios flow cytometer	Ultracentrifuge	Antibody	ANXA5, CD41, and CD235a	(58)
Human platelet-free plasma	BD FACSCanto II	Ultracentrifuge	Antibody	CD71, CD34, CD71, CD235a, and ANXA5	(59)
Human plasma	Beckman CytoFLEX	SEC	Antibody and lipophilic cationic dye	CD9, CD41, and CD42a	(60)
Human plasma from prostate cancer patients	Beckman CytoFLEX	SEC	Antibody	STEAP1	(61)
Glioma cell U373 with EGFRvIII	Beckman CytoFLEX	SEC	Antibody	CD9, EGFR, CD44, and BSG	(55)
DiFi, A431, and human plasma	BD FACSAria IIIu	Ultracentrifuge	Antibody	CD9, EGFR, activated EGFR, and EGFR ligand AREG	(42)
HEK293 or ascites of ovarian cancer patients	Apogee A50	Ultracentrifuge	Antibody and CFSE	EPCAM	(54)
MDA-MB-231, MCF-12A, and human serum	Apogee A50	Ultracentrifuge	Antibody	CD44 and CD47	(62)
PAN02 and mouse plasma	Apogee A60	ExoQuick, Ultracentrifuge	Antibody and CFSE	CD9	(63)

EV, extracellular vesicle; CFSE, carboxyfluorescein succinimidyl ester; SEC, size exclusion chromatography.

Nano-flow cytometry revealed disease-related EV subtypes in the blood, in which the majority of EVs were derived from platelets, erythrocytes, leukocytes, and vessel endothelial cells (60, 61). Arraud et al. found 30% of annexin V-positive EVs derived from platelets and 3% of CD235a-positive EVs derived from erythrocytes in platelet-free plasma (58). In addition, specific antigen-positive EV subtypes, including CD41-, CD42a-, and CD61-positive EVs, are released from platelets (58, 66). Impressively, the configuration of EV subtypes in plasma is affected by pathological conditions such as anemia (59) and cancer (66). Cancer cell-derived EVs are a lower subpopulation than other blood cell-derived EVs but are significantly regulated by cancer status. For example, CD147-positive EVs are significantly upregulated in the blood of patients with colorectal cancer (67), and STEAP1-positive EVs are significantly increased in the plasma of patients with prostate cancer (61). Thus, selective analyses of cancer-specific EV subtypes defined by antigens provide more precise diagnostic information about cancer than bulk EVs.

Although nano-flow cytometry is a powerful tool to provide information on single EV distribution, there are limitations to overcome the noise signal, which is generated by their high sensitivity to detect near 100-nm or lower-sized EVs (49). To discern the EV signal from background noise, fluorescence labeling of EVs is widely used, such as lipid membrane dyes, annexin V (phosphatidylserine affinity), antibodies to specific surface antigens, fluorescent protein-fusion membrane proteins, and chemical dyes (60). In particular, carboxyfluorescein succinimidyl ester (CFSE) is easily used to label almost all EVs (9). This chemical dye is a non-fluorescent compound that easily diffuses into the luminal side of EVs through the membrane and is then cleaved by intravesicular esterases, yielding a highly fluorescent compound. Moreover, after conversion to the fluorescent form, it is retained in the vesicular interior because it is coupled with the free amine group, resulting in the stable retention of the fluorescent signal within EVs. In addition, fluorescent-conjugated antibodies are used to detect specific subpopulations among bulk EVs (9, 42, 54). Notably, the brightness of fluorophores is important in single EV detection owing to their smaller surface area (40), permitting only a lower number of antibodies or fluorophores to be incorporated in an EV than in a cell. Thus, brighter fluorophores should be considered for better resolution between negative and positive EV subpopulations (58). A combination of different fluorophore labeling methods could be applied to detect multiple molecule-positive EV subtypes (9, 55). For example, our group revealed that DNA-containing EVs, labeled by PicoGreen chemical dye, are also positive for EGFR rather than CD63 (9).

Another current challenge of nano-flow cytometry is to detect the smaller exosomal EVs ranging from 30 nm to 100 nm. Beckman CytoFLEX could resolve the polystyrene nanoparticles up to 70 nm by violet laser-based side scatter (violetSSC) (68), and the Apogee flow cytometer could detect 100-nm silica beads by small angle light scattering (54). NanoFCM flow analyzer is able to detect the lower size silica nanoparticles from 40 nm by highly sensitive light-scattering detection (67). Each of the commercially available nano-flow cytometry has demonstrated the sufficient measurement of EVs above 100 nm but has shown limitations to the analysis of lower-sized EVs for accurate detection and precise immunophenotyping (69, 70). Moreover, the multiple smaller EVs could be detected in a single event accompanied by increased fluorescent intensity, known as the swarm effect (71), during the high-speed event acquisition (e.g., over thousands of events per second in Beckmann CytoFLEX) (9). In particular, this swarming is increased in high concentrations of EVs (9, 70) or non-EV particles in blood during the flow (72). To minimize the swarming for a single EV detection in nano-flow cytometry, precise calibration by multiple diluted samples should be necessary to find optimal concentration for the precise measurement of isolated EVs or direct measurement of EV/particles in complex biological fluids such as blood (9, 70, 72).

## Subtype change in EV landscape during cancer progression

Classically, the generation of EVs is considered a mechanism for the removal of unnecessary molecules, as exemplified by the disposal of transferrin receptors during reticulocyte maturation, which led to the initial discovery of exosomes (29, 30). Although this process involves the disposal of unnecessary molecules, it selects specific cargos with complex cellular intrinsic processes driven by mechanisms of endocytosis, membrane budding during MVB maturation, membrane fusion, and exocytosis, representing distinctive functionality. Initially, Raposo et al. discovered the functional role of B cell-derived MHC class II-carrying EVs in activating the T-cell response (73). After this initial discovery of EV functionality, tremendous functional roles of EVs have been revealed in diverse pathophysiological conditions.

Intrinsically, EVs could transfer their molecular contents to the recipient cells by their internalization through phagocytosis, micropinocytosis, endocytosis, or membrane fusion. Taken together, EVs may be either re-utilized as intact EV cargo or degraded by lysosomes, resulting in the loss of their endogenous properties (25). The destination of EVs depends on both uptake routes and EV properties; phagocytes may destroy the uptaken EVs, but macropinocytosis may reutilize the EV components in the endoplasmic reticulum (ER) escaping from the lysosome (74). Importantly, ER localization is EV-specific rather than liposome-specific, which mainly depends on lysosomes (74). Thus, the interaction of cancer cell-derived EVs with target cells, their uptake, and intracellular processing are regulated by both their molecular and physical properties imposed by parental cells and the state of the recipient cells. For example, EVs derived from glioma stem cells are poorly taken up by endothelial cells (75), whereas transformed glioma cells by EGFRvIII favorably internalize their own EVs (55). In addition, activated EGFR coupled with KRAS mutation drives increased EV uptake in pancreatic cancer cells through micropinocytosis (76). This oncogene-mediated EV uptake could be a therapeutic target of a drug delivery system for siRNA delivery in KRAS mutant pancreatic cancer (77). This EV uptake is facilitated by surface proteins in the recipient cells. For example, glypican-1, a GPI-anchored proteoglycan, on the surface of recipient cells promotes the uptake of glioma exosomes (78). Furthermore, EV surface decoration with non-integral membrane proteins known as the EV corona affects EV uptake efficiency. Fibronectin-coated EVs are specifically taken up by mutant RAS-transformed intestinal epithelial cells *via* the heparan sulfate proteoglycan on their surface, where cellular transformation triggered by mutant RAS generates the ruffle structure on the plasma membrane (25).

The secreted EVs interact with the parental (autocrine) or other (paracrine) recipient cells and activate their signaling pathways triggered by receptor activation *via* surface ligands on EVs, enabling collective directional cell migration or proliferation (79). Another target of EVs is the extracellular matrix (ECM) near the parental cells. Cancer cell-derived EVs contain proteases, such as metalloproteinases, which degrade the ECM surrounding the cancer, favoring cancer cell proliferation and invasion (80). Moreover, EVs carrying metalloproteinase ADAM10 play a protective role against bacterial toxins as decoy receptors (81). Malignant transformation massively affects the molecular contents of EVs, such as their bioactive lipids, intravesicular cargos, receptors, ECM proteins, nucleic acids, and metabolites (3). EV proteomic studies indicate that these proteomic alterations during the metastatic transition (2), oncogenic KRAS-derived cellular transformation (82), and proinflammatory cytokine TNF-α stimulation (83) are related to cancer progression and metastatic niche formation (23, 36). Recent reports suggest that EVs derived from cancer cells are heterogeneous with different functionalities according to their subtype (3, 23, 36). For example, glioma cells overexpressing oncogenic EGFRvIII, a constitutively active deletion mutant of the ligand-binding domain, drive the release of pathogenic EV subsets that carry increased oncogenic EGFR and invasiveness-related proteases and adhesion proteins (55) (**Table 3**). Impressively, nano-flow cytometry revealed increased CD44/BSG double-positive EVs in EGFRvIII-overexpressing glioma cells compared with their parental cells, which represents a cellular phenotype with strong co-localization in spike-like invadopodia on the plasma membrane (55). It is known that EGFR-activated signaling pathways *via* RAS-RAF-ERK stimulate the clustering of BSG, CD44, and EGFR on the plasma membrane, forming invadopodia (84). This structure plays a role in cancer invasion by recruiting MMP14 (MT1-MMP) by BSG (84). In terms of the pathogenic effects of glioma CD44/BSG double-positive EVs, CD44 could interact with hyaluronic acid-rich extracellular matrix, and BSG with MMP14 could promote the proteolytic degradation of ECM components, including laminins and collagens (85), suggesting that this EV could favor cancer invasion and metastasis (55). In particular, activation of EGFR seems to partially suppress exosome biogenesis (6) with downregulation of exosomal CD81 and CD82 and to activate ectosomal EV release (55). However, CD9, another canonical EV marker protein, is associated with EGFR-positive EVs; most CD9-positive EVs are EGFR-positive in colorectal cancer cell DiFi (42), and approximately 74% of EGFR-positive EVs are CD9-positive in glioma cancer cell U373vIII (55). Moreover, a recent report suggested that CD9-positive EVs are less positive for CD63 and are directly shed from the plasma membrane (7), representing differential tetraspanin protein equipment in a single EV.

**Table 3:** EV subtypes affected by cancer progression.

Cancer types (oncogene)	Cells	EV subtypes	Methods	Reference
Glioma cancer (EGFRvIII)	U373 and U373vIII (EGFRvIII overexpressing isogenic U373)	CD44 (+), BSG (+), CD81 (−), and CD82 (−)	Nano-flow cytometry (Beckman CytoFLEX)	(55)
Ovarian cancer	Malignant ascites from ovarian cancer patients	EPCAM (+)	Nano-flow cytometry (Apogee A50)	(50)
Breast cancer	Plasma from breast cancer patients	CD47 (−)	Nano-flow cytometry (Apogee A50)	(62)
Pancreatic cancer	Syngeneic C57BL/6 mouse tumor model with Pan02 cells	CD9 (+)	Nano-flow cytometry (Apogee A60)	(63)
Colorectal cancer	Human plasma from colorectal cancer patients	BSG (+)	NanoFCM flow analyzer	(67)

EV, extracellular vesicle.

Liquid biopsy is a promising biomarker source for early diagnosis of cancer metastasis or recurrence, disease progression, and monitoring of treatment response (86). In addition, liquid biopsy-based diagnostic technology is essential in personalized medicine because it offers information as a companion biomarker, enabling tailoring of treatment according to patient-specific mutations and observed responses to drug treatment (86). EV subtype change is observed in the biological fluids of patients with cancer. Malignant ascites from ovarian cancer patients contain an increased EPCAM-positive subpopulation (54). However, the CD47-positive subpopulation is decreased in plasma derived from breast cancer patients (62). In addition, a syngeneic C57BL/6 mouse tumor model with pancreatic cancer Pan02 showed an increased CD9-positive EV subpopulation in mouse plasma, which correlated with tumor growth (63). Thus, real-time analysis of EVs in liquid biopsy with a minimally invasive approach would replace or supplement traditional surgical biopsy.

## Subtype change in EV landscape by cancer chemotherapy

**Figure 3:**
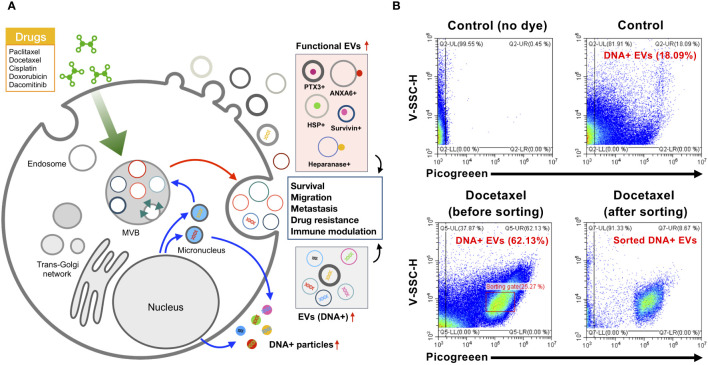
Subtype change of EVs by chemotherapy. **(A)** Chemotherapeutic drugs such as paclitaxel, docetaxel, and dacomitinib markedly alter the release of EVs and their molecular composition with an alteration of protein as well as DNA cargo related to cancer survival, migration, metastasis, drug resistance, and immune modulation in the tumor microenvironment. **(B)** EV sorting by nano-flow cytometry is able to enrich the specific subpopulation of EVs. Docetaxel-treated colorectal cancer cell WiDr showed increased DNA-containing EVs (approximately 62.1%) in comparison to controls (approximately 18.09%). DNA in EVs was labeled by PicoGreen as described in previous research (9). EV sorting by CytoFLEX SRT system (Beckman Coulter) represents the selective sorting of DNA-containing EVs derived from docetaxel-treated WiDr cells. EVs, extracellular vesicles.

Chemotherapies have been widely applied for the effective treatment of most cancers. While the relationship between EV release and chemotherapy is elusive, recent studies have revealed the dynamically regulated EV secretion by chemotherapeutic drugs such as paclitaxel, cisplatin, and doxorubicin, pushing cancer survival, invasion, metastasis, and multidrug resistance for tumor progression (**Figure 3A**) (**Table 4**). This therapeutic stress drives the alteration of the molecular contents of EVs and their release kinetics (96). These transformed EVs are eventually discharged into the body’s circulatory system during chemotherapy. Thus, monitoring these EV cargos or release kinetics could provide information about the status of tumor progression and responsiveness in patients against chemotherapy (97).

**Table 4:** Increased EV subtypes affected by cancer chemotherapy.

Drug name	Cells or biological fluids	Total EV amount	EV subtypes	Functionality	Reference
Paclitaxel	Human liver cancer HepG2	Increased	HSP60 (+), HSP70 (+), and HSP90 (+)	Increased cytolytic activity of NK cells	(87)
Paclitaxel	Human breast cancer cell MDA-MB-231	Increased	CD63 (−) and survivin (+)	Increased survival of fibroblast and breast cancer cells	(88)
Paclitaxel	Mouse breast cancer cell 4T1 (syngeneic mouse model)	Increased	CD9 (−) and ANXA6 (+)	Increased metastasis in mouse tumor model	(26)
Paclitaxel or Eribulin	Human breast cancer cells MDA-MB-231 and HCC1937	Unchanged	CD9 (−) and CD63 (−)		(89)
Docetaxel	Human serum from prostate cancer patients	–	MDR1 (+) and MDR3 (+) in docetaxel-resistant patients		(90)
Docetaxel	Human prostate cancer cell PC-3 (docetaxel-resistant)	Increased	MDR1		(28)
Cisplatin	Human lung cancer cell A549	–	APOA1 (+) and APOE (+)		(91)
Cisplatin or irradiation	Human glioma cell GBM157	–	Splicing factors such as RBM11 (+)	Increased survival of cancer cells	(92)
Anthracycline, taxane, or anthracycline/taxane	Human plasma from breast cancer patients	Increased	TRPC5 (+)		(93)
Doxorubicin	Mouse blood	Increased	CD63 (−)		(94)
Doxorubicin	Human breast cancer cells MDA-MB-231 and MDA-MB-468	Increased	PTX3 (+)	Increased metastasis in mouse tumor model	(95)
Gemcitabine	Human serum from pancreatic cancer patient	–	PLF4 (+)		(96)
Geldanamycin	Mouse glioma cell T103	Decreased	EGFR (−) and EGFRvIII (−)		(97)
Dacomitinib or canertinib	Human epidermal cancer A431	Increased	EGFR (+) and DNA (+)		(98)
Dacomitinib	Human epidermal cancer A431	Increased	DNA (+)		(9)
Bortezomib, carfilzomib, or melphalan	Human myeloma cell CAG	Increased	Heparanase (+)	Increased migration of cells	(99)

EV, extracellular vesicle.

Paclitaxel and docetaxel are widely used chemotherapeutic drugs in a number of cancer types such as breast cancer, lung cancer, ovarian cancer, cervical cancer, and pancreatic cancer (100). Their target is tubulin cytoskeleton defects in cell division, affecting mitotic spindle assembly and chromosome segregation via the stabilization of microtubule formation (100). Lv et al. revealed that paclitaxel stimulates the release of EVs with heat shock proteins (HSP60, HSP70, and HSP90) from liver cancer HepG2 cells, and these EVs activate the cytotoxic activity of natural killer cells by the increased expression of granzyme B (87). Additionally, breast cancer MDA-MB-231 cells showed increased EVs (approximately 1.5-fold) after paclitaxel treatment, in which survivin-carrying EVs were upregulated, although exosomal CD63-carrying EVs were downregulated (88). Survivin is a negative regulatory protein that prevents apoptotic cell death. Thus, survivin-carrying EVs play a role in the increased survival of cancer cells and fibroblasts (88). The downregulation of tetraspanin CD63- or CD9-positive EVs by paclitaxel was also observed in MDA-MB-231 and HCC1937 breast cancer cells (89), implying that non-exosomal EV subtypes could be generated by paclitaxel. This EV subtype change can elicit the distinctive functionality of EVs. For example, Keklikoglou et al. revealed that paclitaxel-treated breast cancer cell-derived EVs significantly stimulated metastasis in cancer mouse models by stimulating annexin A6-positive EV emission, promoting endothelial cell activation and monocyte differentiation in the pulmonary pre-metastatic niche (26). In addition, treatment with anti-myeloma drugs, such as bortezomib, carfilzomib, or melphalan, stimulates EV release containing heparanase, enhancing heparan sulfate degradation and macrophage migration (99). Docetaxel-resistant cancer cells showed an increased release of EVs containing multidrug-resistant proteins, such as MDR1 and MDR3 (28, 90), implying that their chemoresistance is represented by circulating EVs in the patient’s blood. Also, circulating TRPC5-positive EVs are increased in the plasma of breast cancer patients undergoing anthracycline/taxane-based chemotherapy (93). These results indicate that chemotherapy could stimulate the unique types of EVs that show functionality related to tumor progression by modulating the tumor microenvironment, and this information could be used to monitor cancer response to chemotherapy.

Cisplatin is also a widely used drug in various types of solid organ cancers, including colorectal cancer, lung cancer, ovarian cancer, and head and neck cancer (101). Cisplatin is a platinum-based alkylating agent that binds to DNA and inhibits the replication of cancer cells. Non-small cell lung cancer cell A549 treated with cisplatin showed an increased release of EVs containing lipoproteins APOA1 and APOE (91). Furthermore, EVs from apoptotic glioblastoma cells treated with cisplatin or temozolomide promoted the survival and migration of recipient nascent glioblastoma cells (92). In particular, these apoptotic EVs contain spliceosomal proteins, which could be transferred to the recipient glioma cells, affecting their splicing of mRNA and promoting chemotherapy resistance and migratory phenotype (92). Temozolomide is a promising chemotherapeutic drug to alkylate the DNA and is used in treating malignant glioma (102). Likewise, temozolomide could elicit the cyclooxygenase-2 expression in glioma cells, resulting in the upregulation of cyclooxygenase-2-carrying EVs, which have the activity to shift the M2-like pro-tumor phenotype of macrophage (103). Also, temozolomide-treated glioma cells release a different repertoire of small and large EVs, which could stimulate the macrophage to transit the M2-like phenotype with increased cellular expression of IL-6 and IL-10 (104).

Doxorubicin is a potent chemotherapeutic drug that intercalates into DNA, subsequently causing defects in DNA replication by inhibition of DNA polymerase binding, and is used effectively in a variety of cancers, including breast cancer, bladder cancer, and leukemia (105). Mice treated intravenously with doxorubicin generated an increased number of circulating EVs in their blood, in which CD63-positive EVs were upregulated and CD9-positive EVs were not affected (94). Moreover, human breast cancer cell line MDA-MB-231 stimulated by doxorubicin emits proinflammatory glycoprotein PTX3-carrying EVs, which induce cancer metastasis in a mouse tumor model (95).

Dacomitinib, known by the brand name Vizimpro from Pfizer, is a Food and Drug Administration (FDA)-approved irreversible inhibitor of EGFR tyrosine kinase for non-small cell lung cancer. Montermini et al. revealed that dacomitinib dramatically stimulated EV release equipped with phosphorylated EGFR, while cellular phospho-EGFR was inhibited (98). In addition, these EVs contain genomic DNA, which may originate from viable or apoptotic cancer cells that respond to dacomitinib (98). These upregulated vesiculations were affected by caspase activity coupled with exosome biogenesis pathways, implying that tyrosine kinase inhibitors induce EV release to link the traditional exosome and apoptotic EV generation (98). In addition, genomic instability and DNA damage caused by chemotherapy can generate micronuclei, which are involved in the release of DNA-containing EVs (106). Nano-flow cytometry revealed the single EV distribution by EGFR tyrosine kinase inhibitor dacomitinib and canertinib with increased genomic DNA-containing EVs containing EGFR rather than CD63, demonstrating that irreversible blockade of oncogenic EGFR drives the cellular emission of genomic DNA by EVs (9).

DNA-carrying EVs are commonly observed in cancer cells treated with diverse chemical drugs (106). These chemotherapy-induced DNA-containing EV subtypes may be actively released into the tumor microenvironment, contributing to the occurrence of drug resistance, immune modulation, and cancer progression (106). Moreover, a recent study suggested that EVs containing mitochondrial DNA contribute to the development of metastatic breast cancer in which the cancer-associated fibroblast-derived EVs containing mitochondrial DNA promote estrogen receptor-independent oxidative phosphorylation, resulting in escape from metabolic quiescence (107). In addition, it is assumed that EVs containing DNA could be taken up by immune cells, leading to a robust antitumor immune response. DNA-containing EVs from anti-tumor topotecan (DNA topoisomerase I inhibitor)-treated breast cancer cells activate the cytokine release of dendritic cells by cGAS-STING signaling activation in cytosolic DNA-mediated innate immune responses, leading to a more robust anti-tumor immune response (108).

Nano-flow cytometry revealed the nature of DNA-containing EV subpopulations in total EVs responding to the blockade of oncogenic EGFR by EGFR kinase inhibitor dacomitinib showing the release of a wide spectrum of EVs having different sizes and DNA contents (9). This study revealed that only a specific subpopulation of EVs contains DNA, and this composition is affected by chemotherapy, stimulates the apoptotic vesiculation pathway, and releases heterogeneous small EVs containing luminal chromatin (9). However, the investigation of specific DNA-containing EV subtypes is hampered by the limited approaches for the enrichment of DNA-containing EVs. Recent sorting technology coupled with high-sensitivity nano-flow cytometry could enable the enrichment of DNA-containing EV subpopulations. Our study showed that the colon adenocarcinoma cell WiDr released 24.4% of DNA-containing EV subtypes in normal culture conditions, but treatment with docetaxel generated a unique subpopulation of DNA-containing EVs with increased DNA content and violetSSC-H, correlated with vesicular size (**Figure 3B**). This EV sorter could enrich specific DNA-containing EVs, allowing further analyses of individual EV subtypes regarding their characteristics and functionality. Thus, a combination of immunophenotyping, nano-flow cytometry, and selective enrichment of specific EV subtypes could be applied to decode the heterogeneous nature of EVs in the cancer microenvironment affected by chemotherapy in different cancer circumstances.

## Challenges and conclusion

Although the secretion of EVs derived from cancer cells is elevated depending on their progression or chemotherapeutic stress, their relative composition in biological fluids is lower than that of EVs derived from other major normal cells, including blood cells, platelets, and endothelial cells (2). Moreover, other non-vesicular components, including lipoproteins, protein aggregates, and other non-EV particles, make it difficult to detect cancer-specific EV subtypes due to their similar size or density. This substantial challenge can be resolved by the selective analysis of EV subtypes rather than bulk EVs. Affinity isolation of EVs provides a promising enrichment strategy to address specific EV subpopulations using surface vesicular markers such as EpCAM (109) or EGFR (97). For example, Pietrowska et al. enriched cancer-specific EVs from the plasma of melanoma patients using anti-CSPG4 and were able to identify the distinctive proteome of EVs derived from cancer cells (110). Moreover, these identified biomarkers in cancer-specific CSPG4-positive EV subtypes can be directly applied in non-invasive liquid biopsy to monitor cancer progression or response to therapy (110). As described above, recent EV sorting technology can sort specific EV subtypes by nano-flow cytometry with relative enrichment of antigen-positive EVs (42). In addition, it could enrich nucleic acid-positive EVs with nucleic acid-binding fluorescent dyes (e.g., DNA for PicoGreen or RNA for SYTO RNASelect), which is difficult in immunoaffinity-based EV isolations. This single-EV detection technology represents a heterogeneous EV nature with different antigenicity, components, and sizes in the total EV population.

EVs have been increasingly revealed to play roles in the pathogenesis and chemotherapeutic responsiveness of malignant cancer progression. Importantly, they have been considered a capable repertoire in liquid biopsy and diagnostic applications already approved for use in human cancer (86). As discussed above, the traditional total EV analysis meets the considerable intrinsic limitations to understand EV functionality, uptake, and diagnostic potential. These challenges have been re-addressed by single EV analyses, such as nano-flow cytometry, chip-based technology platforms, or single EV imaging (9, 48, 64). These new technologies are capable of revealing the orchestrated and dynamically regulated EV landscape within the EV population in various pathophysiological conditions, especially in cancer. In addition, chemotherapeutic drugs can induce the dynamic change of EV subtypes defined by their molecular cargo, such as proteins or DNA, as well as the increased release kinetics of EVs. Recent studies have revealed that chemotherapy stimulates the unique subtypes of EVs, including DNA-containing EVs derived from viable or apoptotic cancer cells, which represent functional roles in cancer metastasis and immune modulation; however, more detailed knowledge of the effects of chemotherapy on EVs should be further investigated in relation to EV heterogeneity to link single EV biogenesis and intercellular communication in the tumor microenvironment.

## Author contributions

YL: Conceptualization, Data curation, Writing – review & editing. SC: Writing – review & editing. DC: Conceptualization, Data curation, Writing – original draft, Writing – review & editing.
